# Genetic Analysis of Invasive Rats From the Hahajima Islands in the Ogasawara Islands

**DOI:** 10.1002/ece3.74587

**Published:** 2026-10-28

**Authors:** Masafumi Katayama, Kazuto Kawakami, Manabu Onuma, Yusuke K. Kawai

**Affiliations:** ^1^ Biodiversity Division National Institute for Environmental Studies Ibaraki Japan; ^2^ Hokkaido Research Center Forestry and Forest Products Research Institute Sapporo Japan; ^3^ Department of Veterinary Medicine Obihiro University of Agriculture and Veterinary Medicine Obihiro Japan

**Keywords:** D‐loop, Katsuodorijima Island, *Rattus norvegicus*, *Rattus rattus* complex

## Abstract

Based on mitochondrial DNA (mtDNA) sequences, we concluded that the Hahajima Islands are largely inhabited by *Rattus norvegicus* (brown rat), except for Katsuodorijima. The previous study reported that the Chichijima Islands were occupied by *Rattus rattus* complex (RrC) (black rats). We considered that *R. norvegicus* inhabits the Hahajima Islands owing to a combination of factors, such as climatic differences between the Chichijima Islands and the Hahajima Islands, the timing of the invasion by invasive rodents in the satellite islands of Hahajima, and geographical factors. Based on a comparative analysis of the Chichijima individual of RrC, we concluded that RrC II (corresponding to *Rattus tanezumi* ) inhabits Katsuodorijima. The previous study reported that RrC II inhabits Hahajima; therefore, we considered that RrC II migrated from Hahajima to Katsuodorijima by swimming because Katsuodorijima is geographically adjacent to Hahajima and can be reached by swimming. In this study, unique haplotypes (CT6, CT8, and TC7) in *R. norvegicus* at Meijima and Mukohjima, based on the D‐loop analysis, were observed. To the best of our knowledge, these unique haplotypes have not been reported in previous studies; therefore, we considered that these haplotypes originated on Mukohjima and Meijima. Based on this result, although the use of mtDNA data to demonstrate whether migration occurred would be difficult, we suggested that *R. norvegicus* likely did not migrate from Meijima or Mukohjima to other islands. Invasive rodents severely affect the ecosystems of the Ogasawara Islands, including the Hahajima Islands. Predation of Ogasawara Greenfinch (*Chloris kittlitzi*) nests is a representative problem. This study provides helpful information for promoting effective invasive rodent control on the Hahajima Islands.

## Graphical abstract

The black rat ( *Rattus tanezumi* ) inhabits Katsuodorijima Island in the Hahajima Islands. Furthermore, unique haplotypes were observed in Meijima and Mukohjima in the D‐loop analysis.

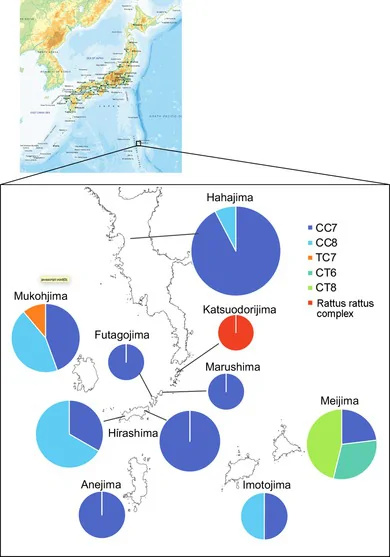

## 1 Introduction

The Ogasawara Islands are subtropical oceanic islands located in the northwest Pacific (Kawakami 2008), and comprise various islands including the Hahajima and Chichijima Islands (Kawakami 2008; Figure 1a). The Ogasawara Islands have never been connected to the Honshu Island, which has resulted in the development of several species that have evolved independently (Kawakami 2008). Consequently, these islands support various endemic species, such as the Ogasawara Greenfinch (*Chloris kittlitzi*) and Bonin flying fox ( *Pteropus pselaphon* ) (Kawakami and Kawaguchi 2021a; Horikoshi et al. 2020; Yoshiyuki 1989). Hence, the Ogasawara Islands are often called “the Galápagos of the Orient.” The unique ecosystem of the Ogasawara Islands, formed by many endemic species and subspecies, has outstanding universal value and hence has been recognized as a World Natural Heritage Site by the United Nations Educational, Scientific and Cultural Organization (UNESCO) (https://whc.unesco.org/document/152476). These islands are internationally considered an area of particular importance for the conservation of ecosystems.

**FIGURE 1:**
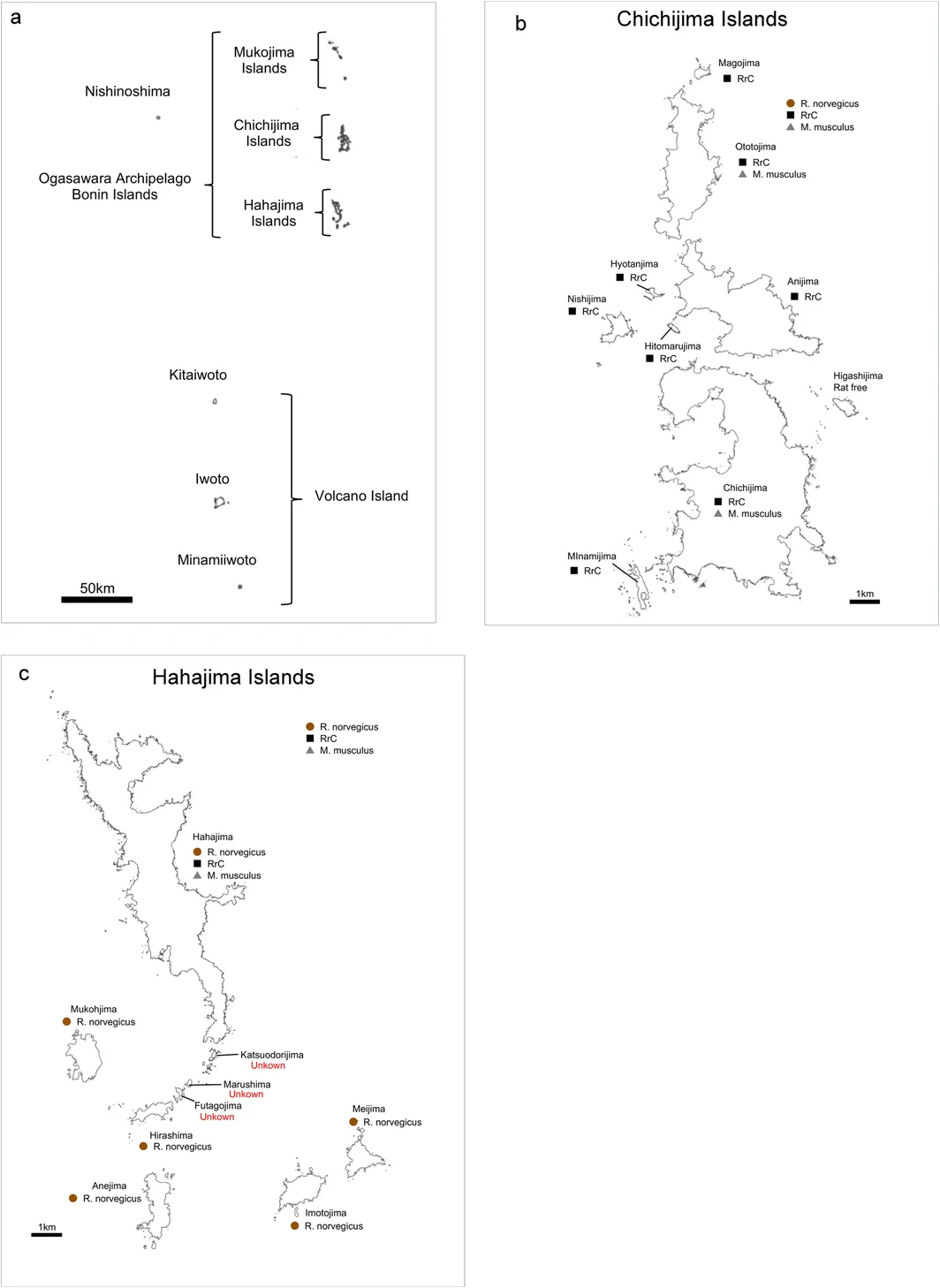
Location of the Hahajima Islands and habitation of invasive rodents in the Chichicjima Islands and Hahajima Islands. (a) Location of the Hahajima Islands in the Ogasawara Islands. Bar shows 50 km. (b, c) Habitation of invasive rodents in the Chichicjima Islands (b) and Hahajima Islands (c). Bar shows 1 km. Brown circles show habitation of *R. norvegicus* , black squares show habitation of RrC, and gray triangles show habitation of *M. musculus* . *Source:* GSI website (https://maps.gsi.go.jp/#5/36.120128/140.097656/&base=blank&ls=blank&disp=1&vs=c1g1j0h0k0l0u0t0z0r0s0m0f1&d=m). According to the Geospatial Information Authority of Japan (GSI) Website Terms of Use, maps are used. In this study, we added graphs and other information to these maps.

Invasive rodents severely impact island ecosystems. They are responsible for the decline and extinction of insular indigenous species and can destroy island ecosystems (Harper and Bunbury 2015; Chiba 2010; Wardle et al. 2009). Brown rats ( *Rattus norvegicus* ), black rats [ *Rattus rattus* complex (RrC)], and house mice ( *Mus musculus* ) are well known invasive rodents. Based on the classification by Aplin et al. (2011), six distinct lineages (I–VI) are recognized within RrC. RrC I corresponds to *Rattus rattus* (2*n* = 38) and RrC II corresponds to *Rattus tanezumi* (2*n* = 42) in the traditional taxonomic framework. RrC II is widely distributed in Japan, whereas RrC I inhabits parts of Japan (Chinen et al. 2005). Recently, Motokawa et al. (2022) reported that RrC IV also inhabits the southern Ryukyus, Japan. The invasive rodents of *R. norvegicus* , RrC, and *M. musculus* were found in the Ogasawara Islands (Kawakami 2019; Figure 1b,c). On the Ogasawara Islands, *R. norvegicus* and *M. musculus* were already confirmed by 1861, whereas RrC was confirmed only in 1930. In the Ogasawara Islands, both RrC I and RrC II inhabit these islands, and these hybrid individuals were generated in these islands (Kambe et al. 2013; Aplin et al. 2011; Chinen et al. 2005). The main distribution area of RrC I and RrC II is different; therefore, the immigration route for the Ogasawara Islands would be different between the two lineages. Originally introduced by human activity, these invasive rodents eventually reproduced and have significantly impacted the ecosystem of the Ogasawara Islands. For example, these invasive rodents have placed considerable predation pressure on land snails, Screw pine (*Pandanus boninensis*), and small seabirds, such as Bulwer's petrel ( *Bulweria bulwerii* ) (Abe 2007; Yabe et al. 2009; Chiba 2010). Therefore, to preserve the ecosystem and biodiversity of the Ogasawara Islands, measures against invasive rats urgently warrant attention.

Invasive rodents are also found on the Hahajima Islands, an island group that comprises the Ogasawara Islands (Kawakami 2019). The Hahajima Islands comprise many islands, including Hahajima, Katsuodorijima, Anejima, and Imotojima. The Hahajima Islands are known as the habitat of *Chloris kittlitzi*, whose occurrence is currently confirmed only on the Hahajima Islands and Minami‐Iwoto (Saitoh et al. 2020; Kawakami 2019).

In this study, we attempted to distinguish rodent species using genetic analysis of the deserted islands of the Hahajima Islands, such as Katsuodorijima, Marushima, Futagojima, Hirashima, Anejima, Imotojima, Meijima, and Mukohjima. Hirashima, Anejima, Imotojima, Meijima, and Mukohjima are breeding areas for *Chloris kittlitzi*. Morphological assessments have revealed that *R. norvegicus* inhabits these breeding areas (Yabe et al. 2017); however, genetic studies have not confirmed the species of these rats. In Katsuodorijima, Marushima, and Futagojima, the rodent species have not been identified as either *R. norvegicus* or RrC (Kawakami 2019; Figure 1c). To establish an effective conservation plan for this species, the rodent species in the habitats should be identified. In this study, we aimed to identify rodent species using genetic analysis of the deserted Hahajima Islands. Furthermore, we aimed to analyze the genetic characteristics of *R. norvegicus* in these deserted islands using the D‐loop region of mitochondrial DNA (mtDNA), which is commonly analyzed for genetic diversity (Sato et al. 2020; Ando et al. 2018). To the best of our knowledge, this is the first genetic analysis of invasive rodents from these deserted islands.

## 2 Material and Methods

### 2.1 Samples of Invasive Rodents

To reduce the number of invasive rodents on Hahajima Islands, administrative organizations conduct rodent eradication programs. In this study, we used rodent samples captured by the Ministry of the Environment and the Forest Agency, Government of Japan, Tokyo Metropolitan Government, and Ogasawara Village under the Eradication program. Additional approval for animal experiments was not required to analyze these samples. Except for the samples from Hahajima and part of the samples from Mukohjima, the rodent samples were collected prior to the extermination efforts. Detailed information is presented in Table 1.

**TABLE 1:** Samples in this study.

Archipelago	Islands name	Collection year	Individual numbers for analysis	Measures to address the problem of invasive rodents
Hahajima Islands	Hahajima	2021	39	While the use of rodenticides and capture using cage traps is being promoted in some areas, the goal is not to eradicate the rodents.
	Katsuodorijima	2022	2	Used the rodenticide (Diphacinone) after sample collection.
	Marushima	2021	4	Used the rodenticide (Diphacinone) after sample collection.
	Futagojima	2021	6	Used the rodenticide (Diphacinone) after sample collection.
	Hirashima	2022	46	Used the rodenticide (Diphacinone) after sample collection.
	Anejima	2022	6	As of now, government agencies have not taken any measures to address the problem of invasive rodents.
	Imotojima	2023	10	As of now, government agencies have not taken any measures to address the problem of invasive rodents.
	Meijima	2023 to 2024	26	Used the rodenticide (Diphacinone) after sample collection.
	Mukohjima	2019 to 2021, 2024	27	Used the rodenticide (Diphacinone) since 2020.

In this study, invasive rodent samples from deserted areas of the Hahajima Islands, including Katsuodorijima, Marushima, Futagojima, Hirashima, Anejima, Imotojima, Meijima, and Mukohjima, were used. These rodents were identified as either *R. norvegicus* or RrC based on their morphology. Invasive rodent samples were collected from the Hahajima Islands, including Hahajima, Katsuodorijima, Marushima, Futagojima, Hirashima, Anejima, Imotojima, Meijima, and Mukohjima. Based on morphological observations, specimens from Hahajima were identified as *R. norvegicus* , whereas those from the other islands were identified as either *R. norvegicus* or RrC. In total, 166 individuals were analyzed: 39 from Hahajima, two from Katsuodorijima, four from Marushima, six from Futagojima, 46 from Hirashima, six from Anejima, 10 from Imotojima, 26 from Meijima, and 27 from Mukohjima (Table 1). Samples from Hahajima were collected in human‐modified environments, whereas those from the other islands were collected in forest, grassland, and bare‐land habitats.

### 2.2 DNA Extraction

In this study, a piece of the tail from an invasive rodent was used. In addition, cultured cells from the ear of Chichijima individuals were used. Based on the morphology, we considered the cultured cells derived from the ear to be fibroblasts. Genomic DNA was extracted from rat tail tissue using the EZ1 DNA Tissue Kit (953034; QIAGEN, Hilden, Germany) following the manufacturer's instructions.

### 2.3 PCR for Confirmation of Species Between *R. norvegicus* and RrC

Species‐specific primers for *R. norvegicus* (AI‐BRT‐0040) and RrC (AI‐KRT‐0040) were purchased from BEX (Tokyo, Japan). For comparison with Hahajima Island samples, we used the DNA of cultured cells from dead *R. norvegicus* and RrC. To detect species differences between the two rat types, we used a PCR assay with a mixture of *R. norvegicus* ‐ and RrC‐specific primers. PCR was performed using 0.5 μL of template DNA using PrimeSTAR Max DNA Polymerase (R045A; Takara). PCR was conducted by pre‐denaturation at 94°C for 2 min, denaturation at 98°C for 10 s, annealing at 56°C for 5 s, and extension at 72°C for 40 s, with 30 cycles of denaturation, annealing, and extension. The PCR products were analyzed using electrophoresis on 2.0% agarose/Tris‐acetate–ethylenediaminetetraacetic acid (EDTA) gels.

### 2.4 Amplification and Sequencing of the D‐Loop Sequence

We amplified the D‐loop sequence of mtDNA using PCR, which was performed using 1 μL of template DNA. The primer sequences used in the study are presented in Table S1. PCR assays were performed using KOD FX Neo (KFX‐201; TOYOBO, Osaka, Japan). PCR was conducted by pre‐denaturation at 94°C for 2 min, denaturation at 98°C for 10 s, annealing at 56°C for 30 s, and extension at 68°C for 45–50 s, with 30–35 cycles of denaturation, annealing, and extension. A portion of each sample was subjected to a nested PCR, which was performed using 0.2 μL of diluted first PCR product (diluted 25‐fold with distilled water). Nested PCR was performed using PrimeSTAR Max DNA Polymerase (R045A; Takara, Ohtsu, Japan). The primer sequences used are presented in Table S1. PCR was conducted by pre‐denaturation at 94°C for 2 min, denaturation at 98°C for 10 s, annealing at 57°C for 5 s, and extension at 72°C for 30 s, with 30 cycles of denaturation, annealing, and extension.

The amplified procedure with PCR stained using BigDye Terminator v3.1 Cycle Sequencing Kit (4337457; Thermo Fisher Scientific, Waltham, MA, USA) with forward or reverse primers of the D‐loop. Primer sequences are presented in Table S1. After staining, samples were sequenced using the Sanger method (3730xl DNA Analyzer; Thermo Fisher Scientific).

### 2.5 Amplification and Sequencing of the Cytochrome b (cyt b) Sequence

In this study, the cytochrome b (cyt b) sequence in mtDNA was examined for species identification of *R. norvegicus* and RrC from Katsuodorijima and Chichijima. PCR was performed using 0.5 μL of template DNA. Table S2 presents the primer sequences used in the study. PCR was performed using KOD FX Neo (KFX‐201; TOYOBO). It included pre‐denaturation at 94°C for 2 min, denaturation at 98°C for 10 s, annealing at 56°C for 30 s, and extension at 68°C for 50 s, with 35 cycles of denaturation, annealing, and extension.

The amplified procedure with PCR was stained using BigDye Terminator v3.1 Cycle Sequencing Kit (4337457; Thermo Fisher Scientific) with forward or reverse primers of the cyt b. Primer sequences are presented in Table S2. After staining, samples were sequenced using the Sanger method (3730xl DNA Analyzer; Thermo Fisher Scientific). The length (bp) of the analyzed fragments is 438 bp (Chichijima) and approximately 600 bp (others).

### 2.6 Sequence Comparison and Phylogenetic Analysis

We used MEGA11 for sequence comparison (Tamura et al. 2021). Multiple alignments were performed using MAFFT version 7; to remove poorly aligned regions, the resulting alignments were trimmed using trimAl v1.5 with the automated1 option (Data S1–, S4), followed by model selection using model finder implemented in IQ‐TREE3. Subsequently, phylogenetic analysis was performed with 1000 ultrafast bootstrap resamplings using IQ‐TREE3, with models selected by model finder (Katoh and Standley 2013; Nguyen et al. 2015). A phylogenetic tree was constructed using MEGA12 (Kumar et al. 2024) or Fig tree 1.4.4. For comparison with the obtained sequence, we used the D‐loop region and cyt b sequences of mitochondrial DNA from *R. rattus* , *R. tanezumi* , and *R. norvegicus* available on GenBank for the determination of rodent species. For the haplotype network analysis, we used the “haplotypes” package in R version 4.6.0 (R Foundation for Statistical Computing, Vienna, Austria) to create a distance matrix using the TCS algorithm that treats indels (indel = 5th), followed by visualization using the “ape” and “pegas” packages. The sequences used for the haplotype analysis are included in Data S3. The accession numbers of the gene sequences obtained from GenBank and their locational sources used for the phylogenetic analyses are presented in Table S3. The D‐loop and cyt b sequences identified in this study are available in GenBank under accession numbers LC927421–LC927595.

## 3 Results

### 3.1 Confirmation of the Rodent Species Between *R. norvegicus* and RrC in Katsuodorijima, Marushima, and Futagojima Using PCR

The aim of this study was to differentiate *R. norvegicus* and RrC in Katsuodorijima, Marushima, and Futagojima. First, we attempted to confirm the species using a PCR assay with *R. norvegicus* ‐ and RrC‐specific primers sold by the manufacturer (Figure 2a). Individuals from Katsuodorijima exhibited a clear band of approximately 100 bp, similar to that in RrC (Figure 2b). The Marushima and Futagojima individuals exhibited a strong band between 200 and 300 bp, which was similar to that of *R. norvegicus* , Hahajima individuals, and Hirashima individuals (Figure 2b).

**FIGURE 2:**
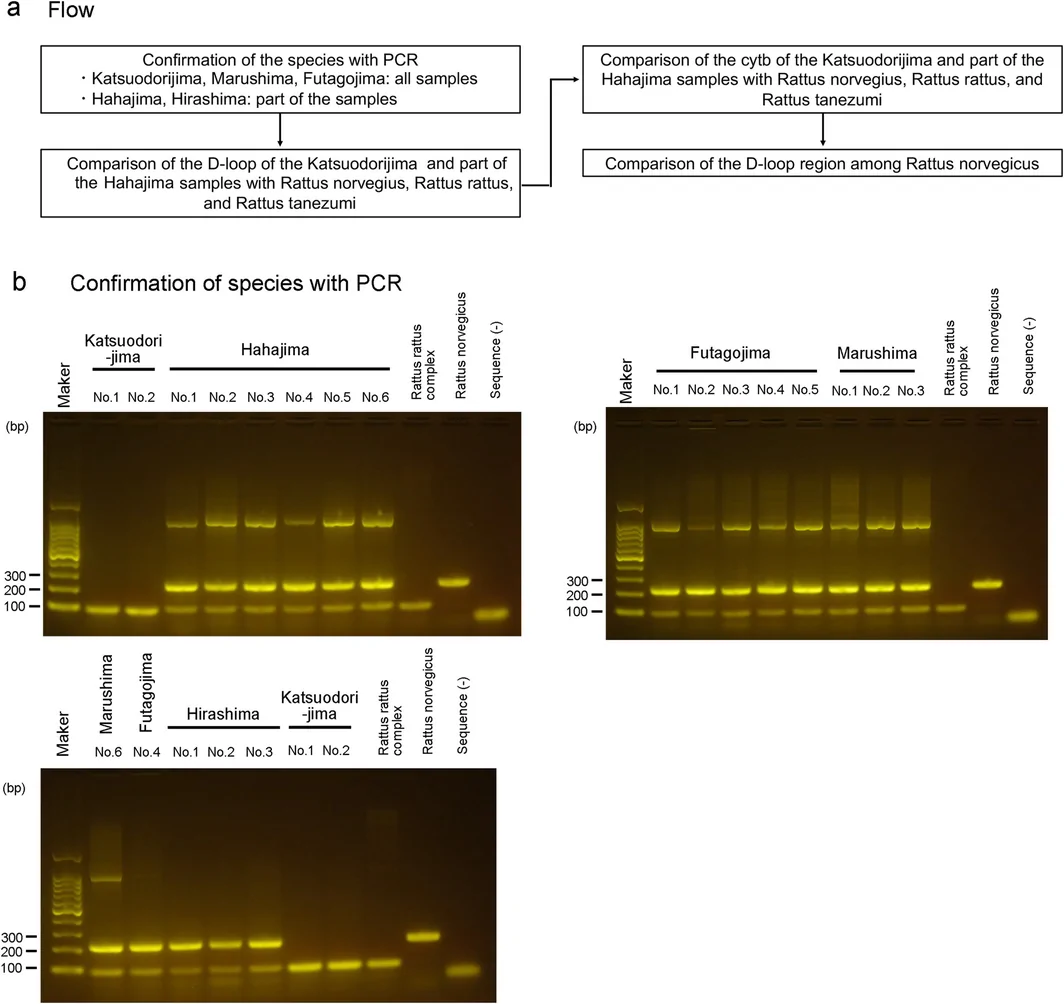
Flow of the study and confirmation of the rodent species between *R. norvegicus* and RrC in Katsuodorijima, Marushima, and Futagojima using a PCR assay using commercial primers. (a) Flow of the study. (b) Confirmation of rodent species by PCR using commercial primers. Electrophoretic images are shown. Upper left: Katsuodorijima and Hahajima; upper right: Futagojima and Marushima; and lower left: Marushima, Futagojima, Hirashima, and Katsuodorijima. The detailed information is provided in the image. Katsuodorijima individuals (No. 1 and 2) in both the upper left and lower images are the same.

### 3.2 Sequence the D‐Loop of Invasive Rodents of Hahajima‐Islands

Subsequently, we compared the nucleotide sequences of the D‐loop in invasive rodents from Katsuodorijima and invasive *R. norvegicus* of Hahajima with those of *R. norvegicus* , *R. rattus* , and *R. tanezumi* (Figure 2a). The sequences of Katsuodorijima individuals in our read D‐loop region sequence were the same as the *R. tanezumi* sequence; however, these sequences were inconsistent with the D‐loop region sequence of *R. norvegicus* or Hahajima individuals (Figure 3a). Our read D‐loop region sequence of *R. norvegicus* from Hahajima individuals was similar to that of *R. norvegicus* (Figure 3a). Subsequently, we constructed a phylogenetic tree of the D‐loop using *R. norvegicus* , *R. rattus* , and *R. tanezumi*, as well as individuals from Hahajima and Katsuodorijima. Individuals from Katsuodorijima formed a single clade with *R. tanezumi* (Figure 3b). The D‐loop sequence of Marushima and Futagojima individuals formed a major clade with *R. norvegicus* (Figure S1).

**FIGURE 3:**
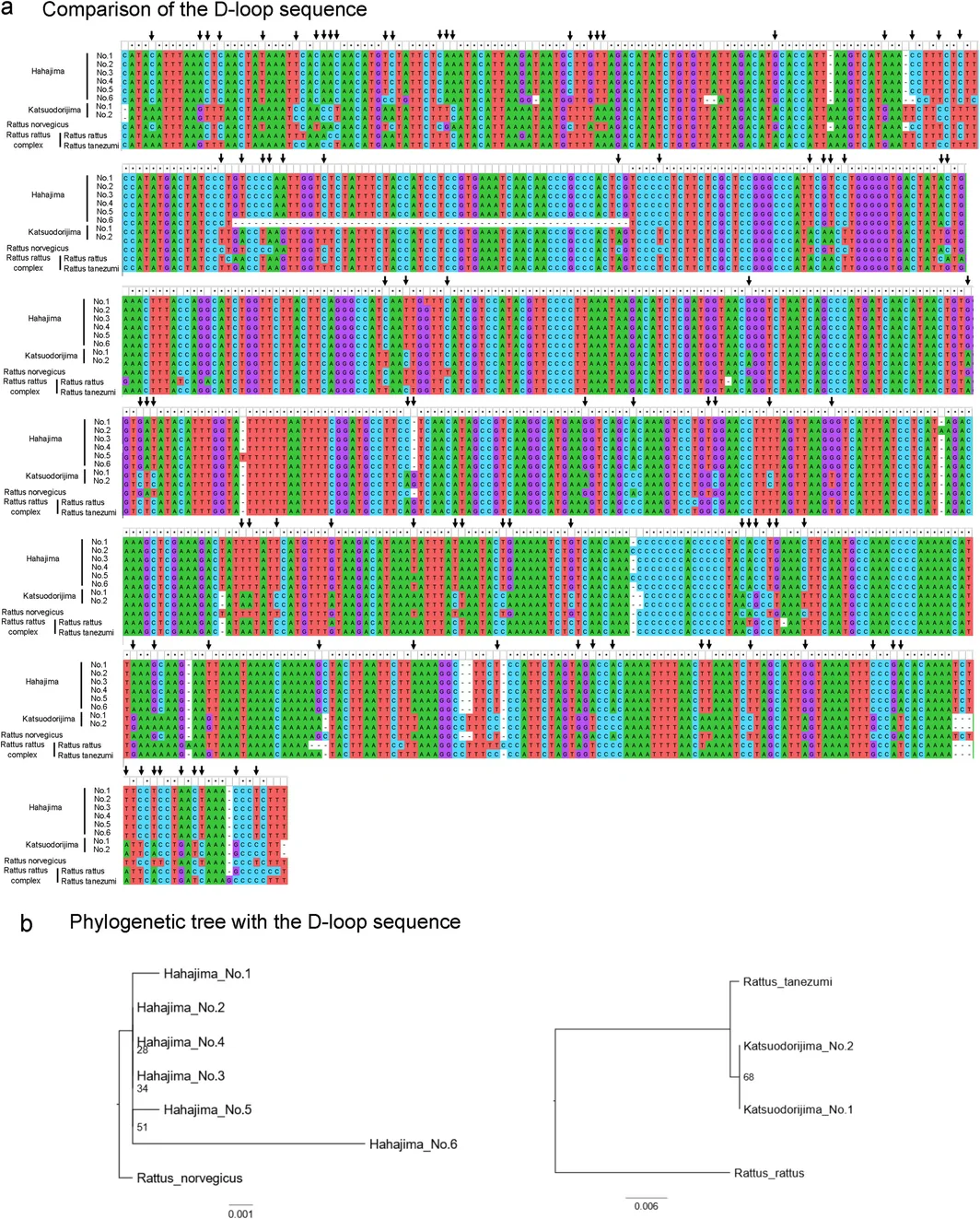
Comparison of the D‐loop sequence of invasive rodents. (a) Comparison of the D‐loop sequences of Katuodorijima individuals, *R. norvegicus* , *R. rattus* , *R. tanezumi* , and some Hahajima individuals. This analysis was performed using MEGA 11 software with Data S1. Arrows indicate the difference in the sequence of Katuodorijima individuals with *R. norvegicus* or Hahajima individuals (95 arrows in total). (b) Phylogenetic tree of the D‐loop using *R. norvegicus* , *R. rattus* , *R. tanezumi* , Hahajima individuals, and Katuodorijima individuals. The left side shows *R. norvegicus* and Hahajima individuals. The right side shows *R. rattus* , *R. tanezumi* , and Katuodorijima individuals. The analysis was performed using IQ‐TREE3 with Data S1. The results were visualized using MEGA 12. Branches with lengths below 0.0001 were collapsed, resulting in multifurcating nodes.

### 3.3 Determination of Species in Katsuodorijima Individuals

We compared the nucleotide sequences of cyt b in *R. norvegicus*, *R. rattus* , *R. tanezumi* , and seven individuals from Hahajima and Katsuodorijima. The cyt b sequences of Katsuodorijima individuals differed from those of *R. norvegicus* and the Hahajima individuals (Figure 4a). In contrast, the cyt b sequences of the Katsuodorijima individuals were identical to those of *R. tanezumi* (Figure 4a). We constructed a phylogenetic tree for cyt b. Similar to the results obtained using D‐loop, Katsuodorijima individuals formed a single clade with *R. tanezumi* (Figure 4b).

**FIGURE 4:**
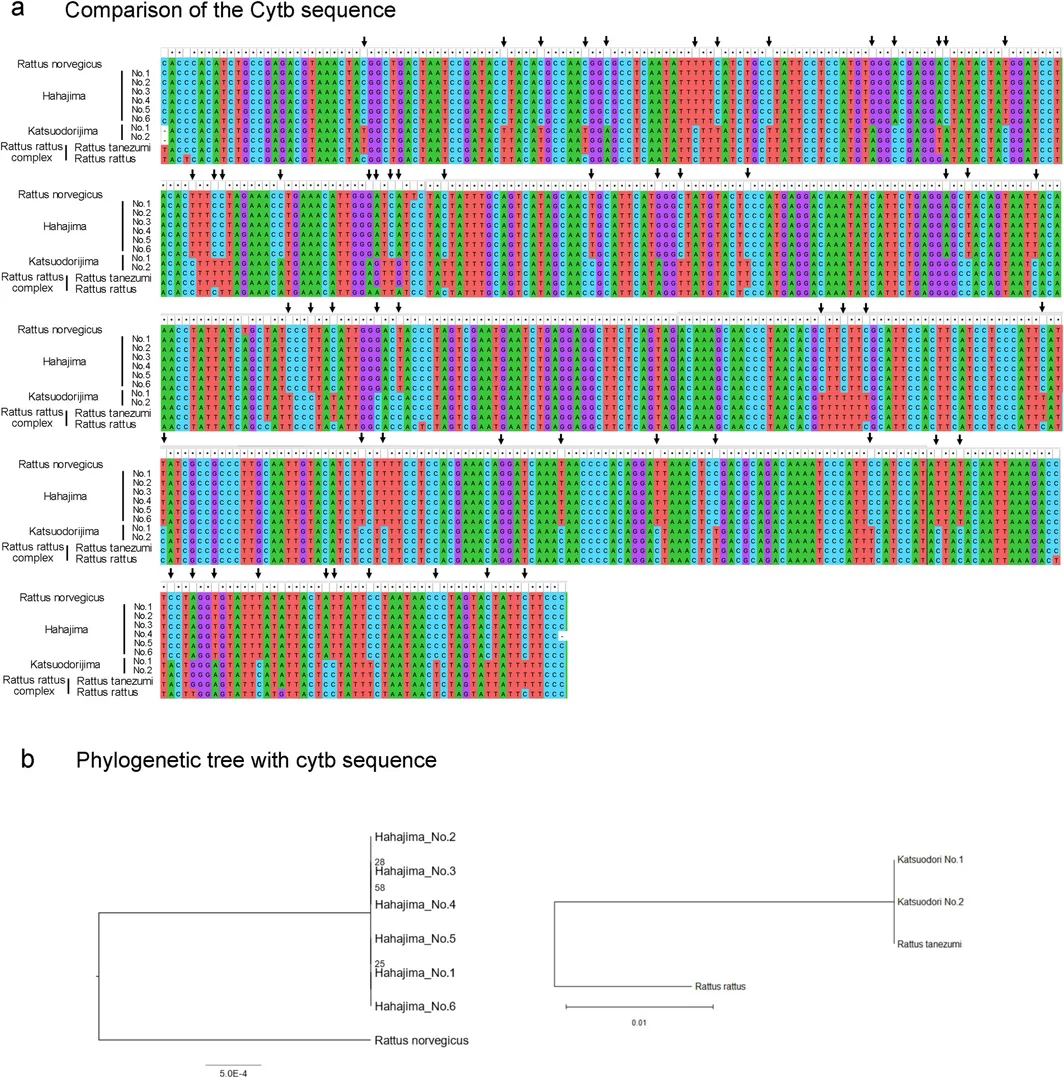
Comparison of the cyt b sequences. (a) Comparison of the cyt b sequences of Katuodorijima individuals, *R. norvegicus* , *R. rattus* , *R. tanezumi* , and some Hahajima individuals. This analysis was performed using MEGA 11 software with Data S2. Arrows show the difference in the sequence of Katuodorijima individuals with *R. norvegicus* or Hahajima individuals (a total of 57 arrows). (b) Phylogenetic tree of cyt b using *R. norvegicus* , *R. rattus* , *R. tanezumi* , Hahajima individuals, and Katuodorijima individuals. The left side shows *R. norvegicus* and Hahajima individuals. The right side shows *R. rattus* , *R. tanezumi* , and Katuodorijima individuals. The analysis was performed using IQ‐TREE3 with Data S2. The results were visualized using MEGA 12. Branches with lengths below 0.0001 were collapsed, resulting in multifurcating nodes.

### 3.4 Comparison of the D‐Loop Sequence of *R. norvegicus* in Hahajima Islands

We compared the D‐loop sequences of *R. norvegicus* from Marushima, Futagojima, Hirashima, Anejima, Imotojima, Meijima, Mukohjima, and Hahajima. We confirmed two SNP sites (C118 and C132) and differences in the number of repeated sequences at one position (Figure 5a). The 118th C sequence was converted to T in some *R. norvegicus* in this area (Figure 5b). The 132nd C sequence was also converted to T in some *R. norvegicus* in this area (Figure 5b). In addition to these SNP sites, 7 sequential C's from the 671st to 677th were converted to 6 or 8 sequential C's in some *R. norvegicus* in this area (Figure 5c). Based on these comparisons, we detected 5 haplotypes (CC7, CC8, TC7, CT6, and CT8) in these individuals. Unique haplotypes, such as CT6 and CT8, were observed in Meijima (Figure 5d,e) as well as in Mukohjima (TC7) (Figure 5d,e).

**FIGURE 5:**
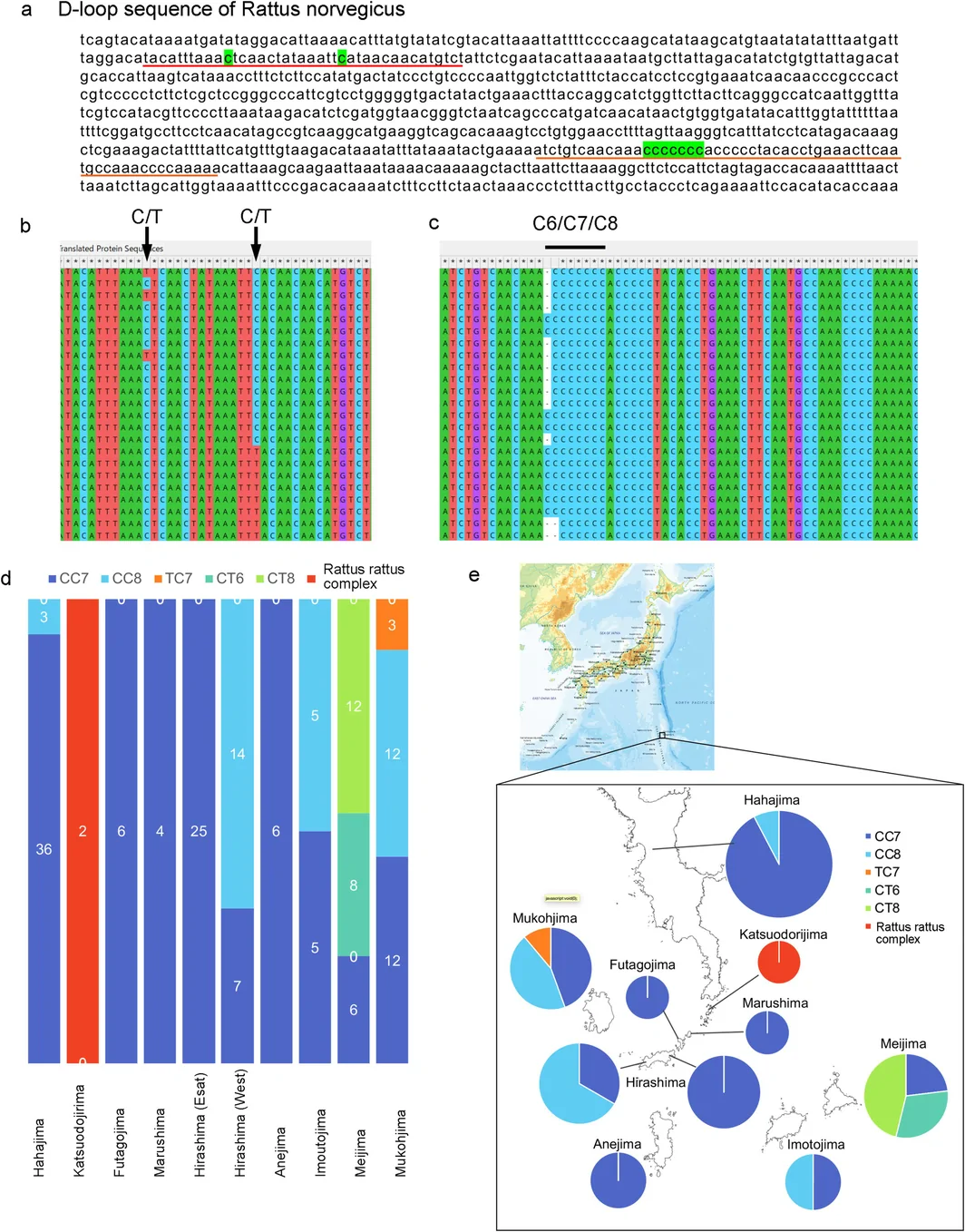
Detection of genetic difference in the D‐loop sequence of *R. norvegicus* in Hahajima Islands and visualization of genetic characteristics of invasive rodents in each Hahajima Island. (a) Genetic differences between *R. norvegicus* from the Hahajima Islands (Marushima, Futagojima, Hirashima, Anejima, Imotojima, Meijima, Mukohjima, and Hahajima). This study confirmed two SNP sites (C118 and C132) and the differences in the number of repeated sequences at each position. These sequences are highlighted in green in the D‐loop sequence of the *R. norvegicus* . (b, c) Comparison of D‐loop sequence of the *R. norvegicus* from the Hahajima Islands (Marushima, Futagojima, Hirashima, Anejima, Imotojima, Meijima, Mukohjima, and Hahajima). Part of the comparison of the D‐loop sequence of the *R. norvegicus* from the Hahajima Islands is shown. Figure 5b corresponds to the red underlined in Figure 5a. Figure 5c corresponds to the orange area underlined in Figure 5a. This analysis was performed using MEGA 11 software with sequences identified in this study (Genbank accession: LC927430–LC927595). (d) Visualization of the genetic characteristics of invasive rodents on Hahajima Island (Katuodorijima, Marushima, Futagojima, Hirashima, Anejima, Imotojima, Meijima, Mukohjima, and Hahajima). The numbers in the graph represent the number of invasive rodents used in the analysis. Five haplotypes were detected: CC7, CC8, TC7, CT6 and CT8. The first C or T is the 118th DNA of Rat D‐loop sequence, the second C or T is the 132nd DNA of Rat D‐loop sequence, and the numbers 6, 7, and 8 are repeat sequences of the 671st to 677th DNA of *R. norvegicus* D‐loop sequence, respectively. (e) Visualization of the genetic characteristics of invasive rodents on Hahajima Island (Katuodorijima, Marushima, Futagojima, Hirashima, Anejima, Imotojima, Meijima, Mukohjima, and Hahajima). In this study, the analysis samples from Hahajima consisted only of *R. norvegicus* , and no other rodents were included. *Source:* GSI website (https://maps.gsi.go.jp/#6/37.046409/136.691895/&base=english&ls=english&disp=1&vs=c1g1j0h0k0l0u0t0z0r0s0m0f0&d=m) (https://maps.gsi.go.jp/#13/26.605488/142.172184/&base=blank&ls=blank&disp=1&vs=c1g1j0h0k0l0u0t0z0r0s0m0f0&d=m). The bathymetric contours are derived from those contained within the GEBCO Digital Atlas, published by the BODC on behalf of IOC and IHO (2003) (https://www.gebco.net). Shoreline data is derived from: United States. National Imagery and Mapping Agency. “Vector Map Level 0 (VMAP0).” Bethesda, MD: Denver, CO: The Agency; USGS Information Services, 1997. According to the Geospatial Information Authority of Japan (GSI) Website Terms of Use, maps are used. In this study, we added graphs and other information to these maps.

### 3.5 Comparison of the D‐Loop Sequence of *R. norvegicus* and RrC in the Hahajima Islands With the Public Database

Next, the D‐loop sequence of *R. norvegicus* and RrC from the Hahajima Islands was compared with the public database. Phylogenetic analysis revealed that the data formed two major clades: one comprising *R. norvegicus* and the other comprising RrC (Figure 6). In the phylogenetic analysis, after alignment, individuals with the same sequence and from the same island of the Hahajima Islands were combined into a single representative sequence (Table S3).

**FIGURE 6:**
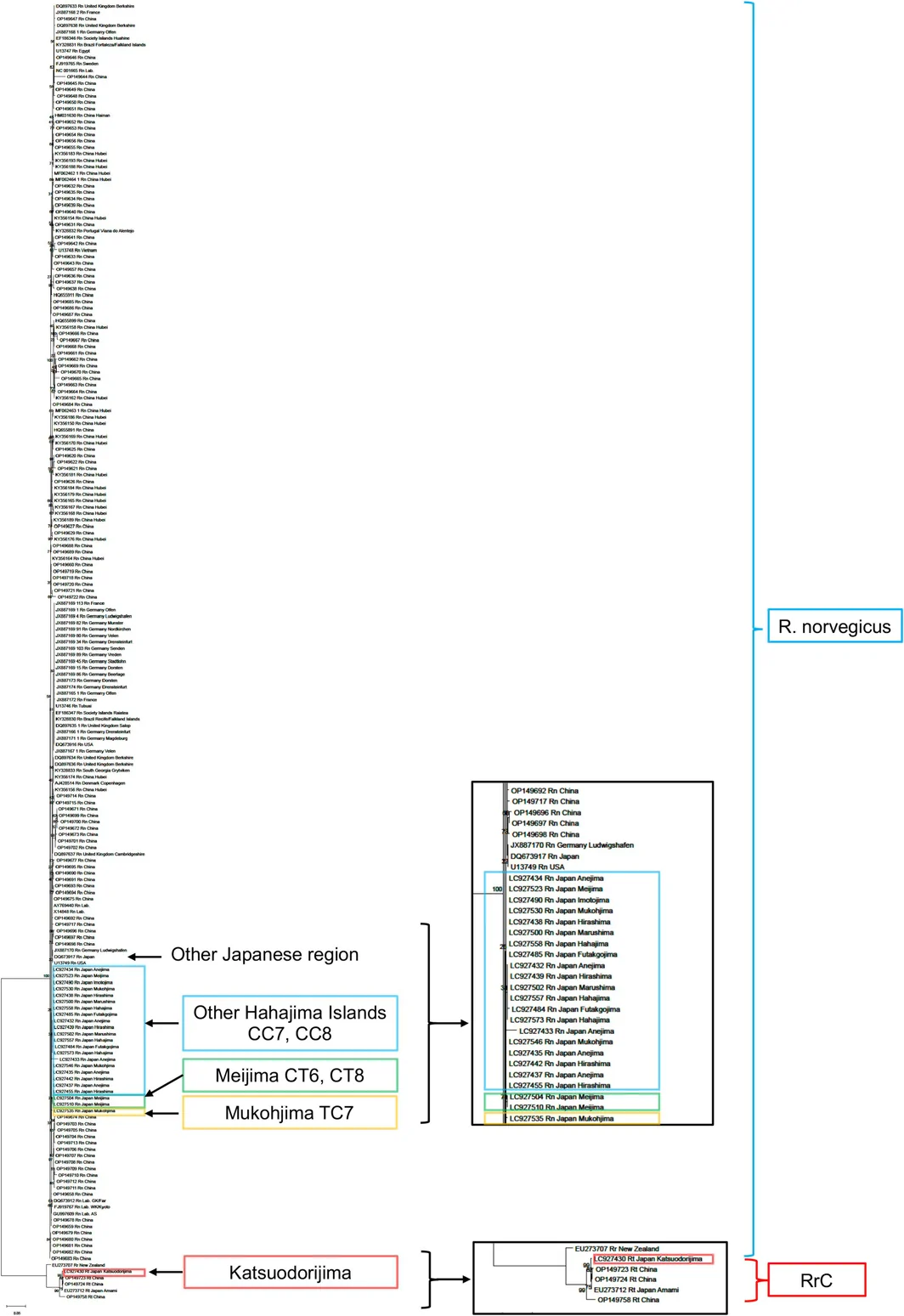
Phylogenetic tree of *R. norvegicus* , and RrC based on the D‐loop gene. The analysis was performed using IQ‐TREE3 with Data S3. The results were visualized using MEGA 12. The values for each node represent the percentage results obtained from 1000 bootstrap resamplings. After alignment, individuals with the same sequence and from the same region were grouped into a single representative sequence (Table S3).

In *R. norvegicus* , all specimens from the Hahajima Islands obtained in this study formed a single clade, supported by a bootstrap value of 37% (Figure 6). We also conducted a haplotype network analysis. The haplotype network analysis showed that all *R. norvegicus* haplotypes are genetically close to one another, and the Hahajima Island haplotypes, including CC7, CC8, CT6, CT8, TC7, formed a cluster in a close position within the network (Figure 7).

**FIGURE 7:**
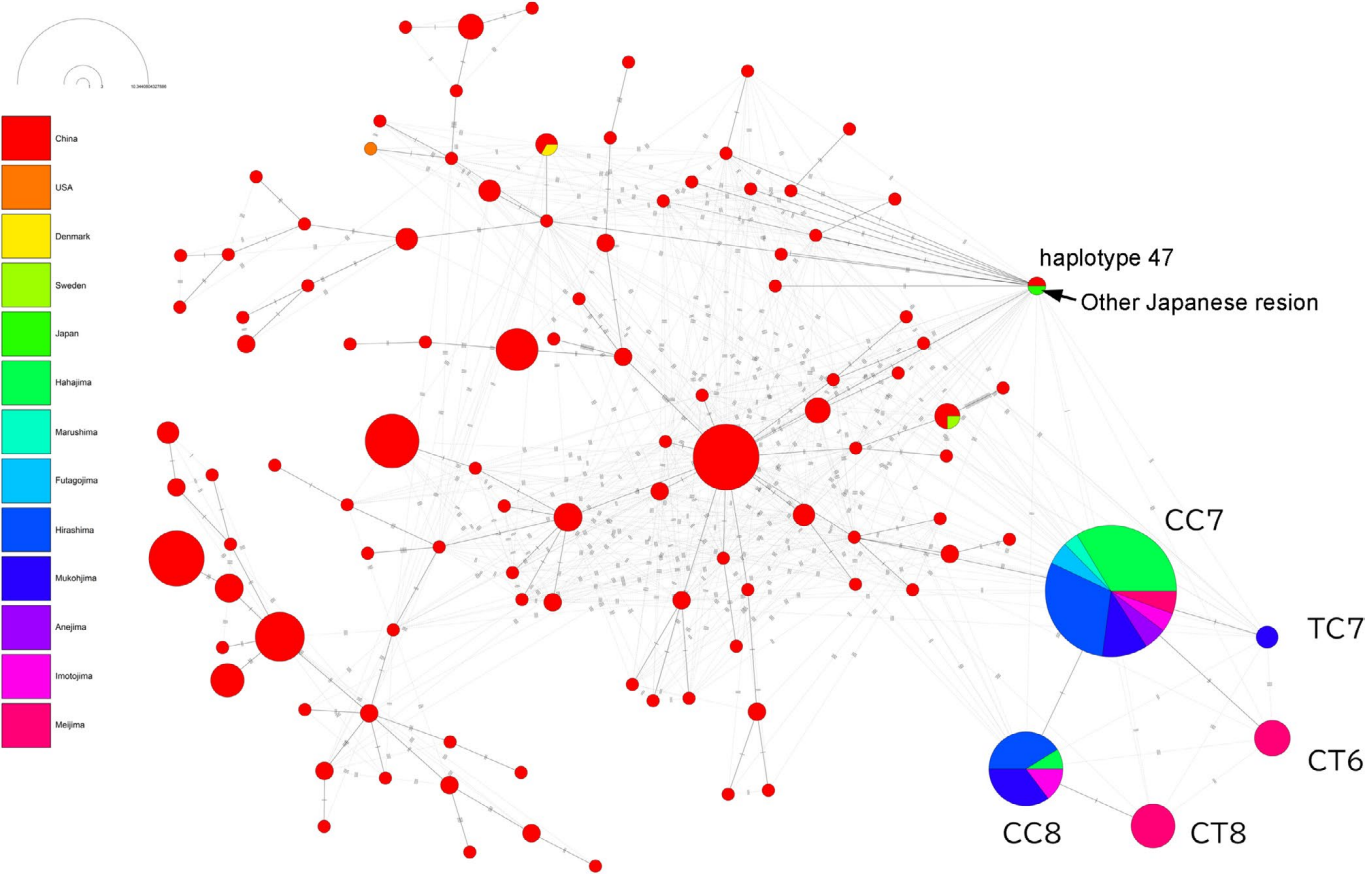
Haplotype network based on the D‐loop gene. Haplotype network analysis was performed using the “haplotypes”, “ape”, and “pegas” packages in R version 4.6.0. After calculating the distance matrix, including indels, using the TCS algorithm, the network was visualized.

### 3.6 Comparison of cyt b Sequences of the Katsuodorijima Individuals, *R. norvegicus* , *R. rattus* , *R. tanezumi* , and the Chichijima Individual of RrC

Subsequently, the cyt b sequences among Katsuodorijima individuals, *R. norvegicus* , *R. rattus* , *R. tanezumi* , and the Chichijima individual of RrC were compared. The sequences obtained for the Katsuodorijima individuals and the Chichijima individual of RrC in this study were consistent with the *R. tanezumi* sequence in the public database (Figure 8).

**FIGURE 8:**
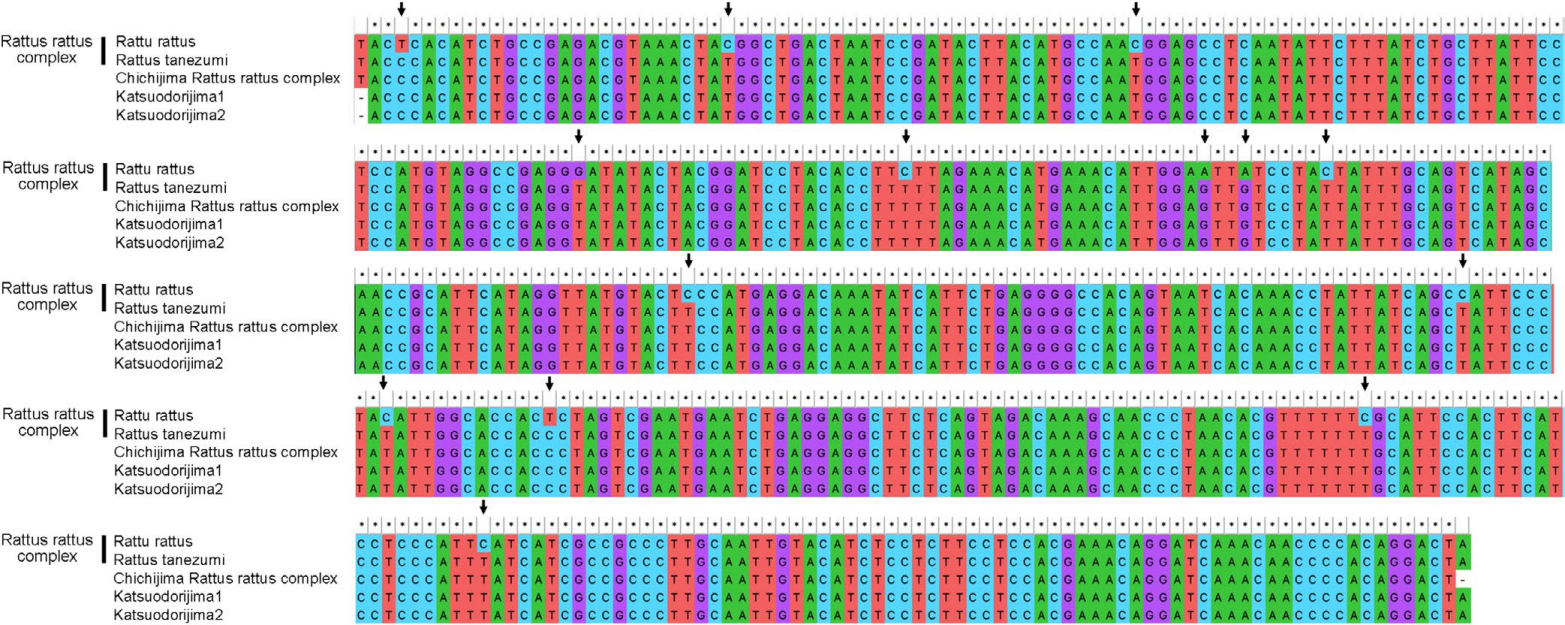
Analysis of the Chichijima individual of RrC. Comparison of the cyt b sequences of Katsuodorijima individuals, *R. norvegicus* , RrC ( *R. rattus* , and *R. tanezumi* ), and the Chichijima individual of RrC. This analysis was performed using MEGA 11 software with the Data S5. Arrows show the difference in the sequence of the Chichijima individual of RrC with *R. norvegicus* or Hahajima individuals.

## 4 Discussion

In this section, we mainly discuss the present distribution of rats in the Hahajima Islands, the PCR analysis, and conservation‐related implications.

First, we discuss the present distribution of rats in the Hahajima Islands. Based on mtDNA sequences, we concluded that the invasive rodent individuals derived from Marushima and Futagojima were *R. norvegicus* . *R. norvegicus* also inhabits Hirashima, Anejima, Imotojima, Meijima, and Mukohjima (Kawakami 2019). Therefore, the Hahajima Islands are largely inhabited by *R. norvegicus* . In contrast to the Hahajima Islands, the Chichijima Islands are primarily inhabited by RrC (Kawakami 2019). This raises the question of why such a difference arose. Yabe and Matsumoto reported that the main difference between Chichijima and Hahajima is the degree of aridity (Yabe and Matsumoto 1982). Notably, Chichijima tends to be arid, whereas Hahajima is humid. Generally, arid areas are a suitable habitat for RrC, whereas *R. norvegicus* prefers moist environments and inhabits areas near coasts and rivers. As one possibility, we considered that these climatic differences might account for the differences in rodent species inhabiting Chichijima and Hahajima. In addition, *R. norvegicus* inhabits Hahajima and its satellite islands. We considered both the role played by the timing of the invasion by invasive rodents and geographical factors in which rodent species inhabit these satellite islands, and the climatic differences between Chichijima and Hahajima. Yabe noted that *R. norvegicus* invaded the Ogasawara Islands before RrC (Yabe 2006). Therefore, we considered that *R. norvegicus* became established on Hahajima before RrC, and subsequently migrated to and established on the satellite islands of Hahajima. Several possible reasons explain why RrC subsequently could not establish a population on the satellite islands of Hahajima. Notably, in addition to the climate mentioned earlier, the already‐established *R. norvegicus* may have prevented the invasion of RrC. Furthermore, we considered that geographical factors also played a major role. Meijima, Imotojima, Anejima, and Mukohjima are located far from Hahajima compared with the distance between Chichijima and the satellite islands of Chichijima; therefore, long distances might prevent the invasion of RrC. In the case of Hirajima, Futagojima, and Marushima, the Marushima Strait, located between Katsuodorijima and Marushima, has strong currents. These strong currents may have also prevented RrC invasion. We therefore attributed the presence of *R. norvegicus* in the Hahajima Islands, including the satellite islands, to a combination of factors.

Subsequently, we discussed how *R. norvegicus* spread throughout the Hahajima Islands. *R. norvegicus* , RrC, and *M. musculus* were already inhabiting Hahajima in the late 1920s. Around the same time, humans lived in Hirashima, Anejima, Imotojima, Meijima, and Mukohjima (Kawakami 2019). Therefore, we considered that *R. norvegicus* was historically introduced from Hahajima to those islands through human activity and subsequently inhabited them. In contrast to Hirashima, Anejima, Imotojima, Meijima, and Mukohjima, there are no records of human habitation on Marushima, Futagojima, and Katsuodorijima. Geographically, Marushima, Futagojima, and eastern Hirashima are adjacent. Our results for D‐loop haplotypes were consistent among these islands (these three areas showed only CC7). Therefore, we considered that *R. norvegicus* would move from eastern Hirashima to Marushima and Futagojima by swimming.

Although mtDNA data are not sufficient to comprehensively elucidate the migration of invasive rodents, in this study, we obtained useful information for their migration from mtDNA sequences. Based on the D‐loop analysis, unique haplotypes (CT6, CT8, and TC7) of *R. norvegicus* were observed at Meijima and Mukohjima. To the best of our knowledge, the unique haplotypes of CT6, CT8, and TC7 have not been reported in previous studies. Furthermore, although those unique haplotypes formed a single clade, individuals exhibiting these haplotypes were genetically closer to individuals from other Hahajima islands than to individuals from other countries or other regions of Japan. Therefore, we considered that this haplotype originated from Mukohjima and Meijima. We therefore considered that *R. norvegicus* likely did not migrate from Meijima or Mukohjima to other islands. Furthermore, CC7 haplotypes were confirmed in Imotojima, whereas CC7 haplotypes were not confirmed in the Meijima individuals. Meijima and Imotojima are adjacent to each other; therefore, *R. norvegicus* was suspected to have moved between them. Individuals likely might not actively swim from Imotojima to Meijima or from Meijima to Imotojima.

This study also shows that haplotype composition differs between the eastern and western sides of Hirashima from mtDNA sequences. Based on these results, we considered that the frequency of genetic exchange between the eastern and western sides of Hirashima may be low. Generally, *R. norvegicus* is considered to have been historically introduced from Hahajima to Hirashima through human activity, as humans previously lived in Hirashima (Kawakami 2019). As one of the hypotheses, CC8 introduced only the western side of Hirashima through human activity, and *R. norvegicus* did not engage in active exchange between the eastern and western sides of Hirashima. Therefore, we hypothesized that CC8 was only observed on the western side of Hirashima. However, we plan to further investigate these hypotheses in the future.

We concluded that RrC inhabit Katsuodorijima based on PCR, D‐loop, and cyt b sequences. Chinen et al. reported that RrC from both Chichijima and Hahajima belong to RrC II in mtDNA, exhibit hybridization between Asian and Oceanian types in the nuclear gene Irbp, and possess a karyotype of 2n = 42 (Chinen et al. 2005). The cyt b sequence of the Katsuodorijima individuals of RrC is fully consistent with that of the Chichijima individual of RrC. Therefore, we concluded that Katsuodorijima individuals are RrC II.

Next, we discuss how RrC II migrated to Katsuodorijima. To the best of our knowledge, there are no records of human habitation on Katsuodorijima; therefore, RrC II was unlikely to have been introduced as a result of human activity. Geographically, Katsuodorijima and Hahajima are adjacent to each other. Hahajima individuals also constitute RrC II (Chinen et al. 2005). Therefore, we considered that RrC II migrated from Hahajima to Katsuodorijima by swimming.

Second, we discuss the PCR analysis. To estimate rodent species in Katsuodorijima, Marushima, and Futagojima, we first attempted species identification using a PCR assay to estimate the types of rodents inhabiting the area. The PCR assay results were consistent with D‐loop sequence readings used to determine rodent species. Generally, although the PCR assay is less sensitive than sequencing, it is more convenient for analysis, because it is rapid and less costly. In particular, this assay might be able to rapidly identify species from hair, feces, or raptor pellets collected in the field without capturing rats. This study suggested that the PCR assay is a useful tool for the determination of rodent species.

In this study, two clear bands, approximately 200 bp and 100 bp, were detected in the analyzed Hahajima, Futagojima, Marushima, and Hirashima individuals. In contrast to these individuals, *R. norvegicus* derived from a laboratory rat only detected the approximately 100‐bp band. Those primers were constructed using mtDNA; therefore, even in the case of a mixed‐breed rat, they will exhibit the genotype inherited from their mother. Therefore, even if a band appears around the 100‐bp mark in a wild *R. norvegicus* , the likelihood that it shows a hybrid between a *R. norvegicus* and RrC is extremely low. Next, we tried to read the sequence of the 100‐bp band. Consequently, the amplified sequence was certainly mitochondrial DNA (Figure S2). This sequence showed a high degree of homology with *R. norvegicus* compared to RrC; therefore, the possibility of DNA contamination from RrC is considered low (Figure S2). One possibility is that a homologous sequence to the primer for the *R. norvegicus* inhabiting the Hahajima Islands may have undergone accidental mutation.

Third, we discuss conservation‐related implications. Invasive rodents have a severe impact on the number of *Chloris kittlitzi* in the Hahajima Islands. Invasive rodents predate the nests of *Chloris kittlitzi* (Kawakami and Kawaguchi 2021b). Consequently, the *Chloris kittlitzi* population has been declining steadily over the years, decreasing to approximately one‐tenth of its size over the past 25 years and halving in the last 10 years (Kawakami and Kawaguchi 2021a). The current population comprises approximately 200 individuals (Kawakami and Kawaguchi 2021a). Therefore, the problem of invasive rodents needs to be addressed urgently.

Although both *R. norvegicus* and RrC contribute to the decline of *Chloris kittlitzi* population, the predation pressure of RrC exceeds that of *R. norvegicus* on tree‐dwelling nests (Foster et al. 2011; Kawakami and Kawaguchi 2021b). This is because *Chloris kittlitzi* builds nests in trees to lay eggs, and RrC are highly skilled at climbing trees (Foster et al. 2011, Kawakami and Kawaguchi 2021b). Therefore, we must prevent the introduction of invasive rodents, particularly RrC, into the breeding area of *Chloris kittlitzi*.

*Chloris kittlitzi* once inhabited the Chichijima and Mukojima Islands, it no longer does so. The primary reason for the extinction of *Chloris kittlitzi* on these islands was considered to be predation by RrC (Kawakami 2019). *R. norvegicus* inhabits many of Hahajima's satellite islands. Because of the lower predation pressure from *R. norvegicus* than from RrC on *Chloris kittlitzi, Chloris kittlitzi* was able to maintain its population on these islands. Furthermore, on small islands, the coexistence of two or more rodent species is difficult; therefore, the presence of *R. norvegicus* on Hahajima islands may have hindered the invasion of RrC (King et al. 2011). Invasive rodents swim across seas. For example, RrC swam 500 m across the ocean, and *R. norvegicus* swam 2250 m across the ocean in New Zealand (Clout and Russell 2006). In addition, RrC have been reported to swim across 750 m of the ocean (Shiels et al. 2014). Therefore, there is a risk of RrC invading from Hahajima and Katsuodorijima to other Islands. Therefore, when implementing eradication programs on Hahajima's satellite islands, reinvasion by RrC should be prevented.

Recent control measures against *R. norvegicus* have contributed to maintaining their low population density on the Islands of Marushima, Futagojima, and Hirashima, indicating the possibility that they may have been eradicated from this region. Consequently, Hirashima is now a major breeding area for *Chloris kittlitzi*; therefore, we must prevent the introduction of invasive rodents, especially RrC, to Hirashima in particular. Because Hahajima, Katsuodorijima, Marushima, Futagojima, and Hirashima are adjacent to one another, there is a risk that RrC will invade Marushima, Futagojima, and Hirashima from Hahajima and Katsuodorijima. If RrC, which has a greater impact on ecosystems than *R. norvegicus* , invade Hirashima, they could cause devastating damage to the breeding of *Chloris kittlitzi*. The rapid eradication of Katsuodorijima individuals is essential for reducing the risk of black rat invasion in Hirashima.

Furthermore, after the eradication of Katsuodorijima individuals, we consider that monitoring the number of rodents in Katsuodorijima will be important for reducing the risk of invasion in Hirashima. Katsuodorijima is adjacent to Hahajima. The distance between Hahajima and Katsuodorijima is approximately 200 m at the closest point. Hahajima is the largest island of the Hahajima Islands and is estimated to be home to many invasive rodents, including RrC. This result suggests that RrC can easily move Katsuodorijima from Hahajima. If RrC were confirmed on Katsuodorijima, their eradication would be required. We propose to designate Katsuodorijima as a line of defense against RrC.

To the best of our knowledge, this is the first genetic analysis of rodents from deserted islands (Katsuodorijima, Marushima, Futagojima, Hirashima, Anejima, Imotojima, Meijima, and Mukohjima). Our findings provide useful information for promoting effective invasive control on the Hahajima Islands.

## Author Contributions

**Masafumi Katayama:** conceptualization (lead), writing – review and editing (lead), writing – original draft (lead), visualization (lead), validation (lead), supervision (lead), resources (lead), project administration (lead), methodology (equal), investigation (lead), funding acquisition (lead), formal analysis (equal), data curation (equal). **Kazuto Kawakami:** conceptualization (supporting), writing – review and editing (supporting). **Manabu Onuma:** conceptualization (supporting), writing – review and editing (supporting), methodology (supporting), supervision (supporting). **Yusuke K. Kawai:** writing – review and editing (supporting), visualization (equal), validation (equal), methodology (equal), formal analysis (equal), data curation (equal), conceptualization (lead).

## Funding

This study was supported by the Environment Research and Technology Development Fund (JPMEERF20254001) of the Environmental Restoration and Conservation Agency.

## Conflicts of Interest

The authors declare no conflicts of interest.

## Supporting information


**Figure S1:** Comparison of the D‐loop sequence in Marushima and Futagojima of invasive rodents. (a) Comparison of the D‐loop sequence of Marushima and Futagojima individuals, *R. norvegicus* , *R. rattus* , and *R. tanezumi* . The analysis was performed using IQ‐TREE3 with Data S4. The results were visualized using MEGA 12. Branches with lengths below 0.0001 were collapsed, resulting in multifurcating nodes. (b) The phylogenetic tree of the D‐loop using the *R. norvegicus, R. rattus, R. tanezumi*, Marushima, and Futagojima individuals. This analysis was performed with IQ‐TREE3 (Data S4). They are visualized with Figtree.
**Figure S2:** Comparison of the 100 bp band of part of Marushima and Futagojima individuals with mtDNA of *R. norvegicus* , *R. rattus* , and *R. tanezumi* . The result was visualized using MEGA 12 with Data S6. Based on the arrows showing sequences, we concluded that the 100 bp band from part of the Marushima and Futagojima individuals is mtDNA from *R. norvegicus* .
**Table S1:** Sequence of primers for amplification and read of D‐loop.
**Table S2:** Sequence of primers for amplification and read of Cytochrome b.

**Table S3:** Information on phylogenetic analyses in this study.

**Data S1:** Supporting information.

**Data S2:** Supporting information.

**Data S3:** Supporting information.

**Data S4:** Supporting information.

**Data S5:** Supporting information.

**Data S6:** Supporting information.

## Data Availability

All the required data are uploaded as Supporting Material.
