# Supplementary material for: Genetic Analysis of Invasive Rats From the Hahajima Islands in the Ogasawara Islands
Source: Ecol Evol. 2026 Oct 28;16(11):e74587. doi: 10.1002/ece3.74587 (PMC13717226; doi:10.1002/ece3.74587)
Supplement: Supplementary file 1 [file ECE3-16-e74587-s001.docx]

**Fig.S1 Comparison of the D-loop sequence in Marushima and Futagojima of invasive rodents.**

a: Comparison of the D-loop sequence of Marushima and Futagojima individuals, *R. norvegicus*, *R. rattus*, and *R. tanezumi*. The analysis was performed using IQ-TREE3 with Data S4. The results were visualized using MEGA 12. Branches with lengths below 0.0001 were collapsed, resulting in multifurcating nodes.

b: The phylogenetic tree of the D-loop using the *R. norvegicus, R. rattus, R. tanezumi*, Marushima, and Futagojima individuals. This analysis was performed with IQ-TREE3, Data S4. They are visualized with Figtree.

**Fig.S2 Comparison of the 100bp band of part of Marushima and Futagojima individuals with mtDNA of *R. norvegicus*, *R. rattus*, and *R. tanezumi*.**

The result was visualized using MEGA 12 with Data S6. Based on the arrows showing sequences, we concluded that the 100 bp band from part of the Marushima and Futagojima individuals is mtDNA from *R. norvegicus*.

**Table S1: Sequence of primers for amplification and read of D-loop.**

**Table S2: Sequence of primers for amplification and read of Cytochrome b.**
